# AnyBio – An easy off-the-shelf masked stereolithography bioprinter conversion combined with radical-scavenging strategies

**DOI:** 10.1016/j.ohx.2025.e00705

**Published:** 2025-09-19

**Authors:** Maj-Britt Buchholz, Nils Bessler, Anne C. Rios

**Affiliations:** a Princess Máxima Center for Pediatric Oncology, Heidelberglaan 25, 3584 CS Utrecht, the Netherlands; b Oncode Institute, Utrecht, the Netherlands

**Keywords:** Open-source mSLA bioprinter, Bioresin optimization, Radical scavenging, Tissue engineering, Microfluidic Chips

## Abstract

Over the last two decades 3D bioprinting has gained momentum to fabricate tissue mimicking constructs serving as tissue models, animal-free drug screening platforms and tissue replacements for regenerative medicine. Recently, the focus has shifted towards the development of light-based bioprinting methods due to its high accuracy and absence of nozzle-induced shear stress on printed cells. However, light-based bioprinting equipment can be costly and photo-sensitive materials difficult to optimize. In this work, we present the conversion of a masked stereolithography printer into a heated, humidified, and easily sterilizable bioprinter, with minimal financial investment (<350 € including purchase of the printer). We provide instructions on how to optimize bioinks and printing conditions and explore novel bioresin additives utilizing cell-friendly photon absorption and radical scavenging strategies. As a proof of concept, we demonstrate the compatibility of our method for the ability of our printer to produce complex and perfusable architectures as well as successful cell printing with high post-printing viability. With this we aim to contribute to accessibility of both hardware and protocols to easily implement bioprinting with minimal barriers.

## Graphical abstract

**Figure:**
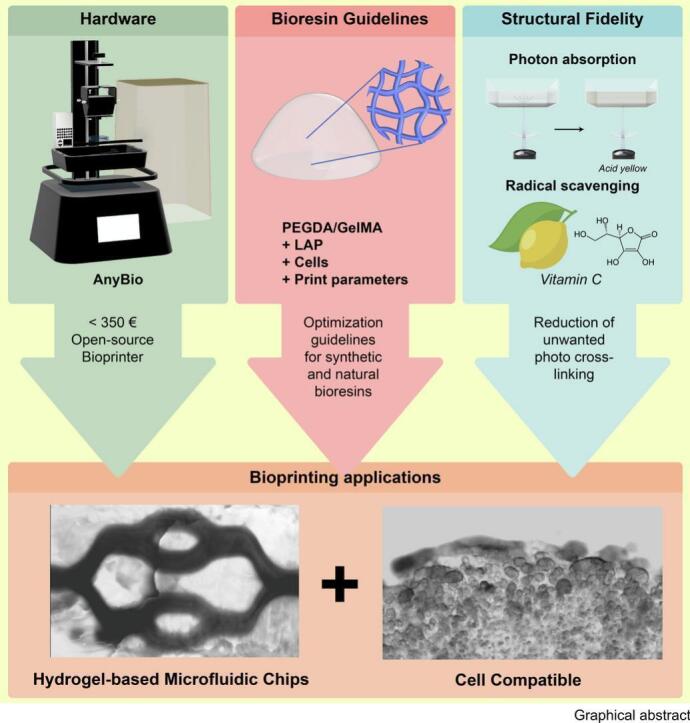

## 1 Specifications table

**Table:** Specifications table of the presented hardware.

Hardware name	AnyBio
Subject area	• Engineering and materials science • Medical (e.g., pharmaceutical science) • Biological sciences (e.g., microbiology and biochemistry) • Educational tools and open source alternatives to existing infrastructure
Hardware type	• Biological sample handling and preparation • Mechanical engineering and materials science • Light-based bioprinter
Closest commercial analog	*TissueRay™ 3D Bioprinter*
Open source license	*GPLv3*
Cost of hardware	∼ 313 € (including purchase of the printer to be converted)
Source file repository	*https://doi.org/10.17605/OSF.IO/HZ4YW*

## 2 Hardware in context

### 2.1 Open-source bioprinting: from extrusion to light-based approaches

Additive manufacturing using biological materials is one of the central focuses of the field of biofabrication. Among these technologies, bioprinting has emerged as a powerful tool for creating 3D structures composed of cells and biomaterials that mimic native extracellular matrices [[1], [2], [3]]. This technological impact has led to advancements on the forefront of e.g. heart[4], lung[5], and bladder models[6]. Much of this progress has been supported by the adoption of accessible, open-source hardware. In recent years, the open science community has contributed to advancements in the biofabrication field by developing a range of bioprinting solutions, from low-cost hardware modifications to complete printer overhauls, that enhance technical capabilities while remaining affordable [5,[7], [8], [9], [10]]. These efforts are typically accompanied by detailed construction guides and protocols based on community best practices, such as embedded extrusion printing [11]. Together, they contribute to a growing ecosystem that expands access to bioprinting technologies for researchers with diverse technical backgrounds [12]. While most open-source conversions have focused on extrusion-based systems, typically adapted from fused deposition modelling (FDM) printers [7,11,13], more recent efforts have turned toward light-based 3D printing and bioprinting techniques such as stereolithography (SLA) and digital light processing (DLP)[10,14,15].

### 2.2 Adapting mSLA printers: emerging conversions towards bioprinting applications

**Fig. 1:**
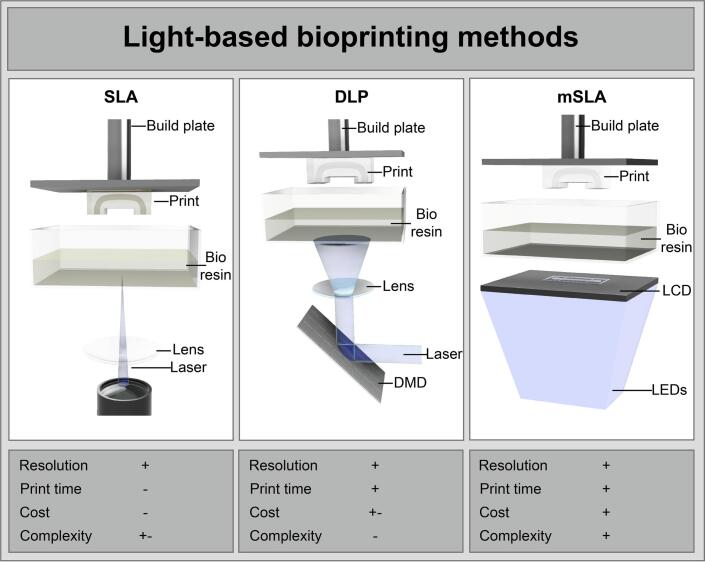
Comparison of SLA, DLP and mSLA printing methods adapted from Qiu et al [17]. In SLA printing (left), a laser draws a pattern of light voxel by voxel onto a layer of photo sensitive resin, while DLP (middle) uses a DMD to project the pattern of one whole layer at once, significantly increasing print speed. Similarly, in mSLA (right) printing an LCD generates a mask letting only the pattern of light of that particular layer through to the bioresin. This technology is cost-effective and complex to build than DLP due to the absence of a DMD while maintaining high printing speeds. Note that only bottom-up approaches are shown, but that top-down approaches follow the same general polymerization principle.

Light-based printing technologies build on the principle of photopolymerization in a layer-by-layer fashion, where patterned light selectively and locally cures a photosensitive resin with high spatial resolution. Pattern generation can be achieved either by guiding a laser beam, as in stereolithography (SLA), or by projecting an entire layer simultaneously using optical masks. In digital light processing (DLP), this mask is created by reflecting light off a digital micromirror device (DMD), while in masked stereolithography (mSLA), an LCD screen modulates light from a 405 nm LED array [16], enabling shorter printing times (Fig. 1**,** adapted from Qiu et al. [17]). In bioprinting, reducing printing times is particularly important to minimise cell exposure to suboptimal environmental conditions, which can compromise viability, metabolic activity and cell function [18]. While SLA, DLP, and mSLA bioprinters are commercially available from companies such as 3D Systems, Cellink, and TissueLabs, they often require a substantial financial investment (∼25.000 €). Affordable open-source alternatives based on DLP technology have been developed, demonstrating the fabrication of intricate vascular and alveolar models using the SLATE bioprinter (∼2700 $) [5]. However, constructing DMD-based systems typically demands a comprehensive technical expertise, limiting accessibility and adoption in less engineering-focused environments.

MSLA printer conversions on the other hand have become especially attractive for research-driven applications due to their high pixel density, low hardware costs, and broad off-the-shelf availability [10,15,[19], [20], [21], [22]] (Fig. 1). These systems have enabled a range of microfabrication applications, including fabrication of microfluidic master molds [21], the optimization of resin formulations for multimaterial microfluidic chips [22], sub-100  µm channel micromoulding using 8 k LCD resolution [20], and the production of lung-on-chip devices [19]. The development of this technology has not gone unnoticed in the bioprinting community. An example of this is the work of Breideband et al., who demonstrated the feasibility of converting an mSLA printer for bioprinting including external temperature regulation, CO2 supplementation, and LCD screen cooling. This enabled the successful printing of bile duct cancer organoids in GelMA and PEGDA constructs using commonly used cell culture slides [10]. Similarly, Kaufmann et al. presented a modular mSLA-based platform with integrated environmental control, including custom temperature and humidity regulation. Their system utilized a modified print build platform within the standard resin vat and demonstrated the fabrication of complex gyroid structures and perfusable channels with diameters of 1.5 mm [15]. Together, these mSLA conversions mark a critical step toward affordable and accessible light-based bioprinting platforms. Other approaches to spatially controlled photocrosslinking, such as multiphoton lithography (MPL) or volumetric bioprinting, have also been explored [[23], [24], [25], [26]], and intriguingly, some of these emerging systems have begun to incorporate LCD screens as photomasks [27].

### 2.3 Controlling photopolymerization in bioresins: photon absorption and radical scavenging

**Fig. 2:**
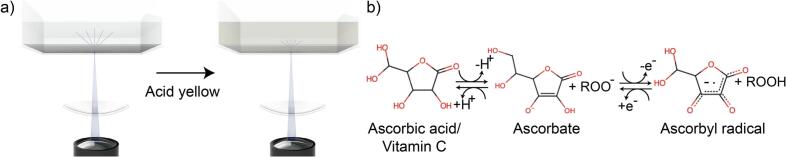
Photo absorption and radical scavenging applied in this paper. a) Acid yellow is supplemented to the bioresin to reduce penetration depth and light scattering. b) Vitamin C supplementation enables scavenging of free radicals through the conversion of ascorbate into a stable ascorbyl radical reducing unwanted photo cross-linking. Chemical structures were drawn using the Chemical Sketch Tool of RCSB Protein data bank^29^. (For interpretation of the references to colour in this figure legend, the reader is referred to the web version of this article.)

While hardware adaptations are undoubtedly important, reaching optimal printing results is critically reliant on bioresin formulation and optimization. Bioresins typically consist of biocompatible, photo-reactive hydrogels such as PEGDA or GelMA, combined with photoinitiators like lithium phenyl-2,4,6-trimethylbenzoyl phosphinate (LAP) or Irgacure. However, achieving high-resolution structures with viable embedded cells requires careful resin optimization beyond the base polymer and initiator concentration. In recent years, two complementary strategies have emerged for improving geometric fidelity while maintaining or enhancing cell viability: photoabsorbers [5,28] and radical scavengers [[29], [30], [31]] (Fig. 2). Photoabsorbers are typically dyes with absorption peaks near the printing wavelength. By limiting light penetration into the resin and scattering into adjacent regions, they reduce curing depth and suppress unwanted polymerization [28,32] (Fig. 2a). More recently, chemical radical scavenging has emerged as an additional layer of control. Since photo-crosslinking relies on radical generation via initiators like LAP or Irgacure, introducing antioxidative compounds into the resin allows excess radicals to be neutralised. This not only improves spatial resolution but can also reduce cellular damage during printing [[29], [30], [31]] (Fig. 2b).

### 2.4 The AnyBio: a cost-effective and user-friendly mSLA bioprinting platform

3D light-based bioprinting offers high-resolution fabrication capabilities critical to advancing tissue engineering and biomedical research. Yet its broader adoption remains limited by the technical barriers of hardware modification and the need for optimized, cell-friendly bioresins. Previous work by Breideband et al. [10] and Kaufmann et al. [15] take important steps towards cost-efficient mSLA printer conversions and make valuable contributions to the open bioprinting community. Yet, practical limitations remain. External environmental control may restrict the printer’s placement, while internal solutions require soldering, wiring, and programming, skills not commonly available in biology-focused labs. Additionally, the use of commercial Ibidi µ-slides as resin vats introduces recurring costs and limits flexibility. In contrast, the absence of a modified vat design constrains parallel printing and increases the risk of cross-contamination when multiple bioresins are used.

Here, we present a cost-effective and easy-to-implement conversion of an off-the-shelf mSLA 3D printer, combined with state-of-the-art bioresin optimization strategies to enable high-quality printing of cell-laden 3D scaffolds. We provide practical guidance on using Vitamin C as a radical scavenger to improve print fidelity and reduce overpolymerization, resulting in the successful fabrication of complex architectures from soft hydrogels. By relying on widely available off-the-shelf components, including the printer, fan-based temperature control, and reusing parts of the printing platform and vat-container, our approach eliminates the need for soldering, wiring or coding, making it accessible to users with minimal technical expertise. The PDMS-based vat design further supports flexible adaptation to different printing geometries and throughput needs. We demonstrate that our integrated hardware and bioresin setup with controllable environmental conditions preserves high post-printing cell viability and metabolic activity. Effectively, this hardware conversion lowers both financial and technical barriers, offering an accessible starting point for researchers and educators exploring light-based bioprinting.

## 3 Hardware description

**Fig. 3:**
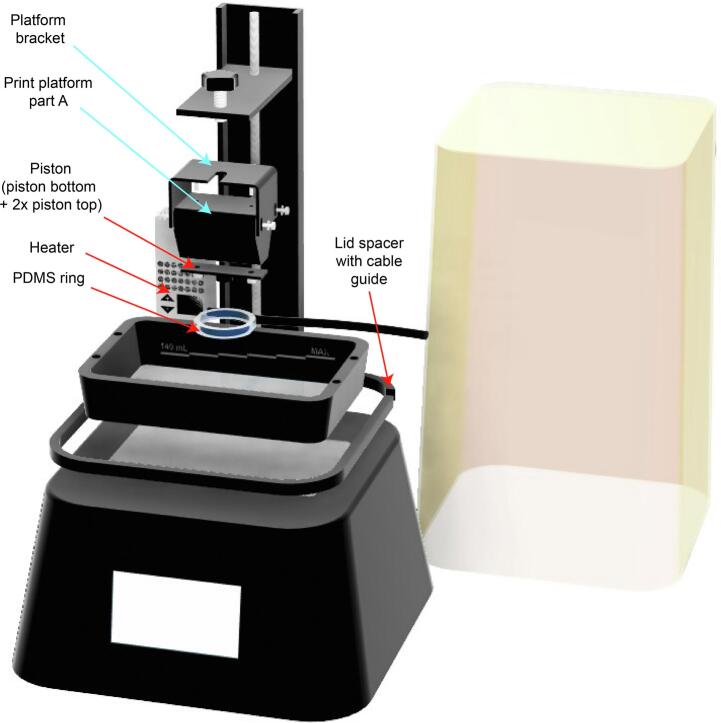
Overview of the AnyBio hardware conversion. The conversion consists of a build plate modification using a CNC milled piston, the vat volume reduction through a PDMS ring and the installation of a heater with the help of a lid spacer allowing for cable guidance. All new parts needed for the printer conversion are indicated with a red arrow. (For interpretation of the references to colour in this figure legend, the reader is referred to the web version of this article.)

The AnyBio consists of a commercially available mSLA printer (Anycubic Photon Mono 4 K) and three modifications (Fig. 3 **red arrows**):

1. Reduction of the resin vat volume to accommodate small volumes of costly bioresins (*PDMS ring*),
2. Complementation of the existing printing platform to fit the new vat dimensions (*Piston*),
3. Addition of heating and humidification of the printers’ inner space to prevent resin evaporation or undesired thermal gelation including cable management (*Heater, Lid spacer with cable guide*).

Since accurate calibration of the printing platform is crucial to obtain high precision results, essential for bioprinting complex and small-scale features, we utilized the printers’ own *platform bracket* and the part of the printing platform that is connected to the platform bracket (Referred to as: *Printing platform part A)* (Fig. 3 **cyan arrows**)*.*

The lower part of the printing platform was replaced by a custom-made *piston*. In order to improve hydrogel adhesion and improve sterility, this piston was developed using 3D printing but finally milled from autoclavable aluminum. Using a 3D printed *Ring mold*, a polydimethylsiloxane (PDMS) ring was produced and attached to the vats fluorinated ethylene propylene (FEP) foil, effectively reducing vat volume to a maximum of 700 µL and allowing the addition of water to the surrounding vat volume, beneficial to ensure humidification (Fig. 3). To ensure consistent temperatures within the printer, we inserted an off-the-shelf fan-based heater and propped up the printers’ lid onto a 3D printed *Lid spacer* allowing guidance of the heaters’ cable. Our aim was to reduce printing costs to a minimum by restricting vat volume to 700 µL. This modification allows prints up to a length of 18 mm. However, the open-source and modular nature of our design allows for quick and easy adaptations to the desired dimensions as well as high-throughput printing when printing smaller items. By attaching several pistons to *printing platform part A* in the future, even higher throughput could be achieved.

The AnyBio displays particular strengths regarding:

• The customizability due to General Public Licensing (GPL).
• Reliable calibration due to the use of original high precision parts.
• The unprecedented low financial burden (124 € for the conversion resulting in less than 350 € total) and required skillset to implement this system in a lab with no prior bioprinting experience.
• The ease of use is guaranteed by detailed operation instructions as well as guidance to optimize new bioresins including control over photothermal effects through the adaptation of the environmental conditions.
• Printing and quantification of complex architectures as demonstrated by printing hydrogel chips containing complex hollow channel networks due to the introduction of radical scavenging compounds in our bioresins.
• The ability to print viable cells as demonstrated through printing of Human Brain Microvascular Endothelial Cells (HBMECs) and Human Embryonic Kidney (HEK) cells with high post-printing viability and metabolic activity.
• The potential to be expanded for parallel high-throughput printing due to an adjustable PDMS-vat approach without consumables e.g. petri-dish, glass or plastic slides.

## 4 Design files

### 4.1 Design files summary

**Fig. 4:**
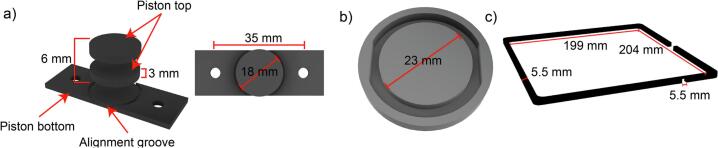
CAD design files used for the hardware conversion. a) The design of the piston consists of a piston bottom and two piston tops with a diameter of 18 mm. b) The ring mold allows for an inner vat diameter of 23 mm. Edges are flattened to avoid collision with the pistons’ bolts. c) The lid spacer contains a 5.5 mm gap for the heater cable to pass through. All files can be found in the OSF repository linked above.

*Piston base* and 2 x *piston top* assembled form the 18 mm *piston (*Fig. 4a*),* which is connected to the original *printing platform part A* to ensure optimal calibration. These were milled from aluminum-covered dibond-polyethylene plates to ensure hydrogel adhesion and shape fidelity in the heated printer space and allow for autoclaving to improve sterility. To reduce vat volume a mold was 3D printed to cast a 23 mm ring from PDMS forming a smaller vat of 700 µL volume within the original vat (Fig. 4b). The ring has flat sides to avoid collision with the mounting bolts of the piston. The 3D printed *lid spacer* allows for the heater cable to be guided outside the printer without the need to drill a hole in the original printer lid (Fig. 4c). All design files can be found in our OSF repository [33] as editable f3d and step files as well as ready-to-print stl files (see Table 1).

**Table 1:** Design file summary.

Design file name	File type	Open source license	Location of the file
Piston bottom	CAD file	GPLv3	OSF Repository[33]: AnyBio-ConversionFiles
Piston top			
Ring mold			
Lid spacer			

## 5 Bill of materials summary

Due to the use of as many original printer parts as possible, the total costs for this hardware conversion including purchase of the printer add to approximately 313 €, see Table 2 below:

**Table 2:** Bill of materials.

Designator	Component	Number	Cost per unit −currency	Total cost − currency	Source of materials	Material type
3D printed parts	*Ring mold*	1	17 cents (€)	17 cents (€)	PVA; 123-3D.nl, Jupiter series, DFV03000	Polymer (PVA)
	*Lid Spacer*	1	49 cents (€)	49 cents (€)	PLA, 123-3D.nl, Jupiter series	Polymer (poly-lactic-acid (PLA) or polyethylene terephthalate glycol (PETG))
CNC milled parts	*Piston bottom*	1	20 cents (€)	20 cents (€)	Alupanel grijs 3 mm RAL 7016, kunststoffplatenshop.nl	Polymer and metal (polyethylene and aluminum)
	*Piston top*	2	5 cents (€)	10 cents (€)	Alupanel grijs 3 mm RAL 7016, kunststoffplatenshop.nl	
Off-the-shelf parts	*Anycubic photon mono 4 K:* • *Printer base* • *Bracket* • *Printing platform part A*	1 each	/	189 €	123-3D.nl	Other
	*Heater*	1	79.99 €	79.99 €	Amazon; Temperature Controller for 3D Printer, 3D Printer Heating Thermostat, Temperature Control and Air Purifier for LCD DLP 3D Printer/	
	*Temperature/Humidity sensor*	1	38.60 €	38.60 €	Shelly H&T Gen3	
Screws and nuts	*M4x8mm bolt*	6	/	∼1.00 €	/	Metal (Stainless steel)
	*M4 washer,*	4				
	*M4 nut*	2				
Other	*PDMS Ring (resulting from Ring mold)*	1		3.25€	PDMS (Dow, SYLGARD™ 182 Elastomer kit)	Polymer (PDMS)

## 6 Build instructions

### 6.1 CNC milling and 3D printing of the required customized parts

The piston consists of three parts: the *piston bottom* and 2 x the *piston top*.

CNC mill (Sainsmart Genmitsu 3040 CNC) *piston bottom* and 2 x *piston top* from 3 mm polyethylene covered with black aluminum (kunststofplatenshop.nl; Alupanel grijs 3 mm RAL 7016) using a 2 mm end mill suitable for low melting plastics (Stepcraft; End mill set “low melting plastics” (11706)) at 10.000 rpm spindle speed, 125 mm/min federate and 0.25 mm maximum stepdown.

*Attention: If CNC milling is not available, the piston can be 3D printed as one single part. However, be advised to use a polymer with a high heat distortion temperature (eg. PETG or Nylon) to avoid inaccuracies through thermal deformation (as observed for PLA) when heating the inside of the bioprinter. Further, make sure the material is stable upon repeated ethanol exposure or heat resistant enough to be autoclaved. Note that for PLA and PETG printed pistons we observed poor hydrogel adhesion when using GelMA-based bioresins, while PEDGA-based bioresins adhered well to both PLA, PETG and aluminum. If GelMA-based bioresins are desired, metalization of printed parts such as summarized by Perera et al.* [34] *can potentially induce the same adhesion-promoting effect.*

*Warning: CNC milling of aluminum leads to very fine metal particles that are dangerous to inhale and may irritate the eyes. Wear a mask as well as goggles when milling or place the CNC into an enclosed space.*

3D print the *ring mold* from 1.75 mm PVA at 0.15 mm layer height at 200 °C printing- and 55 °C bed temperature (Prusa Slic3r).

*Note: The ring mold can also be produced from non-dissolving polymers such as PLA or PETG to be re-used. In that case, care must then be taken to take the PDMS ring out carefully to not cause any ruptures, which would result in the bioresin leaking outside of the vat.*

3D print the *lid spacer* from PLA at 0.2 mm layer height at 215 °C printing- and 60 °C bed temperature (Prusa Slic3r) (we used black PLA at 1.75 mm diameter).

### 6.2 Molding and mounting of the PDMS ring to reduce vat volume (Fig. 5)

Mix PDMS (Dow, SYLGARD™ 182 Elastomer kit) component A and component B at a ratio 10:1 and fill into the mold. Allow the PDMS to solidify overnight at 37 °C. Dissolve the mold under stirring using warm water (50 °C) for approximately 1 h to retrieve the *PDMS ring*. Measure the distances (61 mm from the left and right side and 30 mm from the front and back side) of the vat and mark them. Apply PDMS to the bottom side of the *PDMS ring* and place it between the markings onto the FEP foil of the vat. Place a weight on top of the ring and allow the PDMS to solidify at 37 °C for 24 h.

**Fig. 5:**
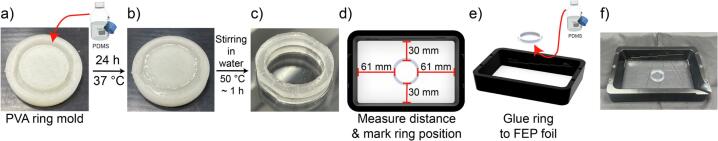
Production of a mini vat within the printers’ own vat. a) 3D printed ring mold. b) Ring mold filled with PDMS. c) Retrieved PDMS ring. d) Positioning of the PDMS ring within the VAT. e,f) Mounting of the PDMS ring and final result.

### 6.3 Assembly and mounting of the piston into the new printing platform (Fig. 6)

Glue the two *Piston tops* onto the *piston bottom* using Loctite^TM^ super glue and using the *piston bottoms’* groove to guide proper alignment. Screw the assembled piston to *printing platform part A* using 2xM4x8 mm bolts and 2xM4 nuts. Attach this new printing platform to the printers’ platform *bracket* using the four M4 bolts (From now on referred to as *calibration bolts*) and washers delivered with the printer. Mount the assembly onto the printer using the printers’ platform securing knob.

**Fig. 6:**
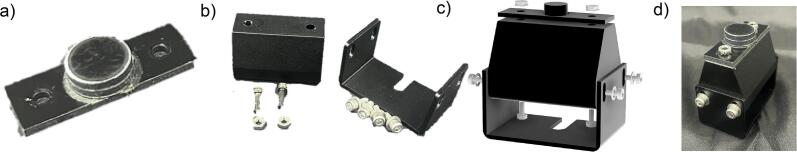
Assembly of the new build plate. a) CNC milled assembled piston. b) Original printer parts and bolts: Printing platform part A and Printer bracket. c, d) Schematic and picture of whole printing platform assembly from CNC milled piston and original printer parts.

### 6.4 Addition of a heater to maintain stable temperature and humidity conditions (Optional) (Fig. 7)

Place the *heater* in the back left corner of the printer. Glue the lid to the *lid spacer* so that the gap for cable guidance is on the back right site. Arrange the *heater* cable to go in front of the z axis of the printer and place the lid-lid-spacer assembly so that the cable runs through the cable guide hole.

**Fig. 7:**
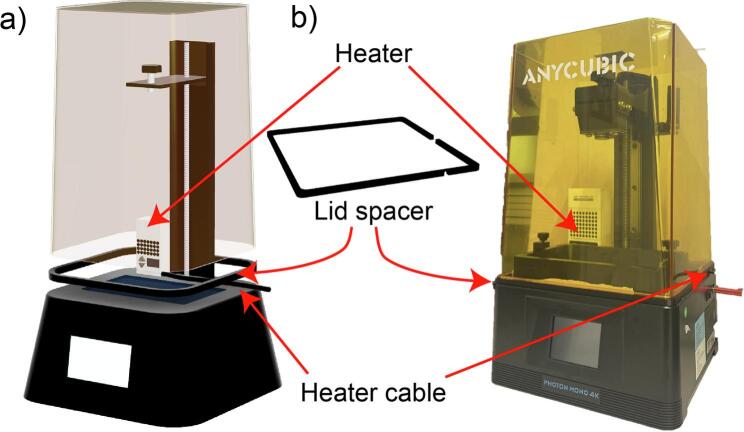
Installation of the heater. a) Schematic and b) Image of the heater, spacer and cable arrangement.

*Note: If prints are conducted with non-temperature-sensitive materials such as PEGDA and without living cells, heating and humidification might not be necessary.*

## 7 Operation instructions

### 7.1 Experimental design/Before printing

#### 7.1.1 CAD modelling (Fig. 8)

Before printing complex models, it is advised to use benchmark models to ensure the print quality is as desired with the specific bioresin used. In our OSF repository (Folder: AnyBio-BioprintableFiles [33] we provide several benchmark models to examine the quality of positive and negative printing features as well as a simple and a complex chip model. After optimizing printing conditions for your specific bioresin on the benchmarks, adjust the dimensions of your CAD models according to the achieved print quality and export to stl or obj format.

**Fig. 8:**
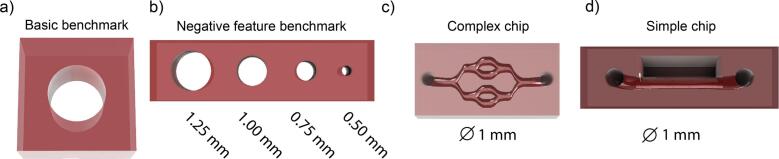
*Provided CAD models for bioprinting.* a) Square with hole to assess overall range of printing parameters by sharpness of edges and openness of the circle. b) Negative feature test containing channels of 0.5, 0.75, 1 and 1.25 mm diameter. c) Chip with 1 mm diameter channel next to a pocket. d) Complex chip with 1 mm channel diameters and branching architecture.

#### 7.1.2 Slicing

Slicing of 3D objects was performed using free-to-use Lychee Slicer 5 (v. 5.4.300) software, in order to control burn-in and transitional layers if necessary.

Place imported objects in the middle of the digital printing platform. The following slicer settings were used throughout the trials with variations in exposure time depending on the material and model used (Table 3):

**Table 3:** Print settings.

Burn In Layers		Normal Layers	
No. of Layers	70	Layer Thickness	50 µm
Exposure Time	15–25 s	Light-off Delay	1
Transition Layers Count	0	Exposure Time	15–25 s
Lift Distance	1 > 2 mm	Lift Distance	1 > 2 mm
Lift Speed	60 > 60 mm/m	Lift Speed	60 > 60 mm/m
Retract Speed	30 > 30 mm/m	Retract Speed	60 > 60 mm/m

Note: We effectively used the burn-in layers as normal layers. Depending on the differences and resins used, we noticed delamination when transitioning from burn-in to normal layers.

### 7.2 Sterilization of all components (only necessary when operating with cells)

Ethanol clean all printer parts (*careful with electronic connectors*) and let them air dry in a sterile flow cabinet. UV expose all objects for 20 min inside the flow cabinet. The printing platform can alternatively be autoclaved.

### 7.3 Calibration

Since overall printing platform height was kept the same as for the non-converted printer, the platform can be calibrated according to the regular Anycubic photon mono 4 K instructions.

Briefly, loosen the four *calibration bolts* and drive the printing platform to its home position. In the home position tighten the four bolts evenly and with reasonable force and set the z position to zero in the printers’ menu.

*Attention: Make sure to loosen the screws all the way before driving the printing platform to the home position to avoid tilting.*

*Warning: In this step you will drive the piston inside the reduced vat volume. If the PDMS ring was not mounted precisely, the piston can get stuck on the edge of the ring, possibly leading to damage of the printer's z axis. Observe this process carefully and, if in doubt, stop the process by powering off the device. Adjust the PDMS ring position and try again.*

*Note: We found that an offset of 500 µm worked best for printing hydrogels. You can set this by either driving the z axis 500 µm up before setting the new zero or by setting a z offset in the slicer settings.*

### 7.4 Pre-warming and humidification (Fig. 9)

**Fig. 9:**
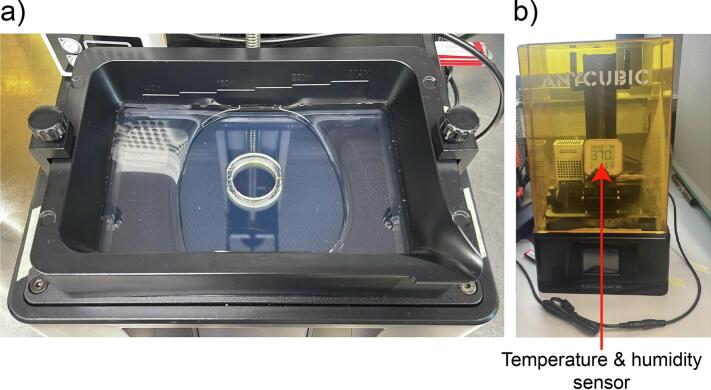
Obtaining humidified and heated printing conditions. a) The outer vat is filled with sterile water. b) The temperature and humidity sensor is placed inside the printer and temperature and humidity are monitored over time.

Add Water into the part of the vat not enclosed by the PDMS *ring (*Fig. 9a*)*. Insert the temperature and humidity sensor inside the printer (Fig. 9b). Let the printer pre-warm until a stable temperature and humidity are reached.

*Attention: It is best to perform assembly of the print platform as well as calibration before this step, so that the printer only has to be opened very shortly to add the bioresin.*

### 7.5 Hydrogel preparation

To dissolve all components start by preparing a Phosphate Buffered Saline (PBS) solution without magnesium and calcium. Dissolve potassium chloride (KCl, 0.20 g/l), potassium phosphate monobasic (KH2PO4, 0.20 g/l), sodium chloride (NaCl, 8.00 g/l) and sodium phosphate dibasic (Na2HPO4, 1.15 g/l) in deionized water (Milli-Q). In case the solution is used for sterile printing, sterile filter it through a 0.2 µm filter and optionally add 1:500 primocin (Invitrogen, ant-pm-05) or another microbial reagent to prevent infections.

Dissolve PEGDA (Sigma Aldrich, mol wt 700, 455008-100ML) in PBS at room temperature at a final concentration of 6 % w/v, 0.4 % w/v LAP (Lithium phenyl-2,4,6-trimethylbenzoylphosphinate, Sigma Aldrich, 900889-1G), 0.033 % w/v Tartrazine (Acid Yellow 23, TCI Chemicals, F0088). Additionally add Vitamin C/L-ascorbic acid (Sigma Aldrich, A92902-25G) in the desired final concentration (from stock: 1 mM, final: 1–10 µM).

Dissolve GelMA (Rousselot, X-Pure GelMA average MW = 160 kDa, degree of methacrylation 80 %) in PBS at 37 °C at a final concentration of 7.5 % w/v, 0.4 % w/v LAP (Lithium phenyl-2,4,6-trimethylbenzoylphosphinate, Sigma Aldrich, 900889-1G), 0.033 % w/v Tartrazine (Acid Yellow 23, TCI Chemicals, F0088). Keep the solution at 37 °C until used. Always prepare the solution the same day as used.

### 7.6 Cell preparation

Culture Human Brain Microvascular Endothelial Cells (HBMEC) (Innoprot, P10361) as described by the supplier. Dissociate a confluent well using StemPro Accutase (Gibco, A1110501) for 5 min at 37 °C and 5 % CO_2_. Add 1 ml of medium (Here: Lonza, EGM-2 Endothelial Cell Growth Medium-2 BulletKit, C-3162) to stop the reaction and perform cell counting and viability assessment using an automatic cell counter or a burker chamber. Next, centrifuge for 3 min at 300 rcf, remove the supernatant and resuspend the cells in the desired amount of PBS for the final concentration of the hydrogel.

### 7.7 Bioprinting

In case of working with a GelMA-based bioresin, keep the environmental temperature at 33 °C prior to printing (see Fig. 14 **a-d** for effects of bioresin heat-build up). Pipet the volume for the desired construct plus some excess volume inside the PDMS ring in the prewarmed bioprinter. Choose the desired construct via the user interface of the 3D printer. Manually adjust any printing parameters necessary (e.g. exposure time) while the piston is lowering.

*Attention: Avoid creating air bubbles while dispensing the hydrogel into the vat as this will interfere with the printing process.*

*Note: The retract speed can influence the printing process, depending on the viscosity of the material with slower retract speeds (e.g. 0.5 mm/sec) being preferable for viscous bioresins (e.g. GelMA).*

### 7.8 Retrieval and culture of bioprinted constructs

Post-printing, detach the construct using a wetted scalpel or razor blade. Immediately transfer the object into a container (e.g. 6-well multiwell plate) with prewarmed (37 °C) PBS or cell culture medium. In case the construct contains negative features (e.g. channels), flush them with warm PBS or cell culture medium to remove any unpolymerized resin using a pipette. To remove any dye residuals, leave the object submerged until the PBS or medium is saturated. We recommend exchanging the liquid every 2 h and leaving the object overnight with fresh liquid. Continue with the exchange, until successfully discolored. The time until full removal of the dye may vary depending on the object size. In case that the object needs to be translucent immediately after printing, one can utilize a 405 nm lamp to discolor the object. Note that this discoloring step will not remove the dye. Refresh media every other day or as often as your cell type requires.

*Attention: Remove the print immediately after the print has finished and wash three times with 37°C PBS to avoid unwanted post-crosslinking (dark curing) or drying out of the sample.*

*Attention: Remove the construct carefully to avoid disrupting it. If you experience difficulties, add a few hundred micrometers extra to the bottom of the design so in case of rupture, no functional parts of your design are affected.*

### 7.9 Analysis of cell viability post-printing

#### 7.9.1 Casting of hydrogel samples as non-printed controls (Fig. 10)

Pipet 15 µL hydrogel-cell suspension per well into a StemCell embedding sheet. Use a UV lamp and expose samples for an appropriate amount of time (Here: 10 s; depending on the strength of your lamp). Remove casted samples from the embedding sheet using a sterile spoon and transfer them into wells containing cell culture media.

**Fig. 10:**
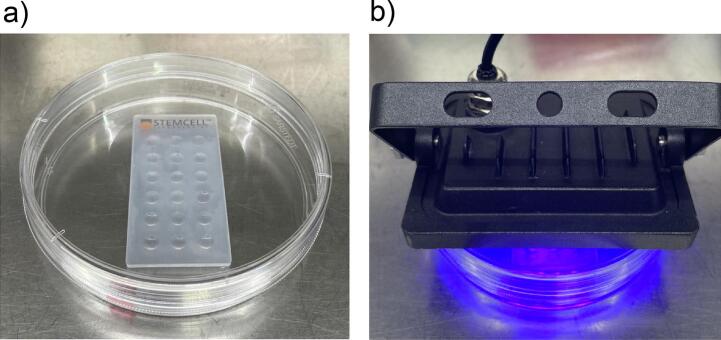
Manual casting of hydrogel samples.

#### 7.9.2 Live/Dead staining

For immediate control of the cell viability after printing, transfer your printed and casted samples into adequate medium containing 1:500 calcein AM (Invitrogen, C1430) and 1:3000 TO-PRO-3 (Invitrogen, T3605) following the manufacturer's instructions. Briefly, incubate for 20 mins at 37 °C and 5 % CO_2_. Wash the samples with fresh medium and image your samples. Quantify the percentage of dead cells within your samples. Compare the viability of your printed samples to casted samples and cell viability before printing/casting to evaluate the short-term influence of the printing procedure on cell viability.

#### 7.9.3 Alamar Blue assay

To assess long-term viability and metabolic activity of printed constructs, perform Alamar Blue (AB) assays using phenol red–free DMEM/F-12 medium (SILAC Advanced DMEM/F-12 Flex, Gibco A24943-01) supplemented with 1  g/L D-(+)-glucose, 1 % GlutaMAX (Gibco, 35050–038), 1 % MEM Non-Essential Amino Acids (Gibco, 11140–035), and 1 % Penicillin–Streptomycin (Gibco, 15140–122). A 10 % (v/v) solution of Alamar Blue (Thermo Fisher, DAL1025) can be prepared using this medium. Incubate with each sample with 300 µl of AB solution at 37 °C for 2 h on e.g. days 2, 4, and 6 after printing. Subsequently, transfer 200 µl of the supernatant into a glass-bottom 96-well plate (PerkinElmer Pheno plate 96-wells) and measure fluorescence using a CLARIOstar Plus plate reader (BMG Labtech) at an excitation wavelength of 560 nm and an emission wavelength of 590 nm in bottom-read mode. We recommend to include constructs with and without cells to normalize for Alamar Blue absorption by the gel, as well as medium-only blanks for background correction.

#### 7.9.4 Analysis of print accuracy

Flip printed chips containing channels or features on the respective side, e.g the channel opening facing up or on the bottom side. With a microscope (e.g. M205 from Leica), take images of the constructs in brightfield mode. If the microscope supports fluorescent excitation, submerge the printed hydrogels in a corresponding fluorescent dye (e.g. Cy3.5 or FITC) for at least 2 h, followed by multiple washes using PBS, to reach better contrast when imaging. In Image J/Fiji or microscope-specific software (e.g. LAS X, ZenBlue), measure the diameters of all resolved channels or features, mark non-resolved channels or features with *NA.* Calculate the percentage deviation of the channel diameters from the intended diameters of the CAD design.

Example for a channel:

Intended diameter: 1.25 mm.

Measured diameter: 1.3 mm.

Percentage deviation: ((1.3/1.25)-1)*100 = 4 %.

Calculate the mean percentage deviation and plot the distribution of deviations or absolute measurements with the mean of your replicates in a violin plot, bar graph or boxplot using ggplot2. We provide an exemplary analysis and plotting script in our repository: AnyBio-Analysis. Negative values of deviations are considered underpolymerized and positive values are considered overpolymerized.

## 8 Validation and characterization

### 8.1 Characterizing basic printing parameters, environmental conditions: heating, energy dose, photoinitiator and photo absorption and printer conversion

**Fig. 11:**
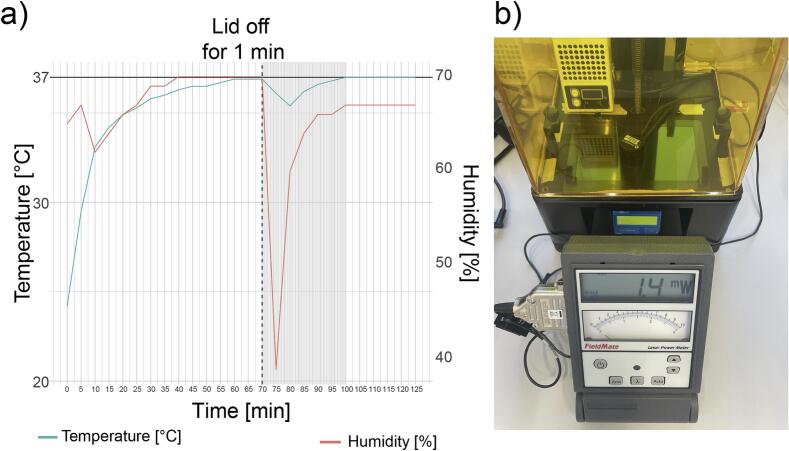
Temperature and light intensity inside the AnyBio. a) Temperature & humidity curve over prolonged time and after taking the lid off for 1 min. b) Measurement of light intensity.

As many bioinks such as GelMA as well as cells are responsive to temperature and humidity changes, we installed an off-the-shelf heater and filled the outer vat with water. Temperatures took approximately 60 min to fully stabilize to 37 °C. Humidity levels already reached a steady state of 70 % after 40  min. To simulate realistic handling operations (e.g. addition of bioresin), we removed the lid entirely for 1  min. Both temperature and humidity levels dropped shortly after but stabilized again within 30  min after closing the lid, highlighting the need to wait for the printer to equilibrate to ensure consistent printing conditions. However, temperature levels only dropped by 2 °C, which is within a reasonable range to maintain GelMA in a liquid state (Fig. 11a).

*Attention: We observed that when the heater was set to 37 °C, the inner space did not exceed 34 °C, and only when set to 40 °C was an internal air temperature of 37 °C reliably achieved. However, due to accumulated light exposure during printing, the bioink itself can heat up beyond the surrounding air temperature. These photothermal effects are context-dependent (eg. environmental temperature, print time, geometry, printer-specific factors). We therefore recommend validating bioink conditions experimentally to determine the equilibration time and actual bioink temperature in their specific lab setup.*

Using the optical detector OP-1 VIS with a 7.9 mm radius (Coherent FieldMate, #1098297) and the Coherent Damage threshold calculator [35], we measured 1.4 mW laser power output of our printer through the FEP foil at 405 nm (Fig. 11b). Using the manufacturers online calculator together with parameters of the sensor being used (OP-1 VIS), the laser power (mW) was converted to the power density (W/cm^2^) using the following settings: Optical Power, Beam profile: Flat top, Beam shape: Circular, Diameter: 7.9 mm, Power: 1.4 mW. This results in a final power density of 2.86 mW/cm^2^. Depending on the utilized exposure time per layer (s, in this study: 15–25 s) one can calculate the power density to energy dose (mJ*s/cm^2^), resulting in accumulated energy doses of 42,9 – 71,5 mJ*s/cm^2^, which is in comparable ranges previously reported for DLP printing (∼61mJ*s/cm^2^) of GelMA[28].

**Fig. 12:**
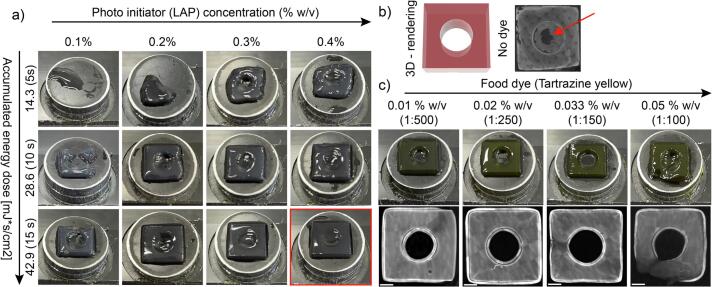
Strategy to optimize photoinitiator and −absorber (PEGDA). a) Elucidating the right combination of LAP concentration and exposure times. b) Comparison of the original CAD file (left) and the print (right) shows a closed hole (red arrow). c) The supplementation of tartrazine yellow allows open circular structures. Prints were executed at an accumulated energy dose of 57.2 mJ*s/cm^2^ (20 s). Scale bar = 2 mm. (For interpretation of the references to colour in this figure legend, the reader is referred to the web version of this article.)

Next, we investigated the practical effects of varying exposure time and LAP concentration in PEGDA-based formulations to establish a first set of parameters for effective polymerization control. While low LAP concentrations yielded underpolymerized prints even at high exposure times, concentrations of 0.3–0.4 % w/v and 15 s exposure time were found to produce the sharpest edges (Fig. 12a). We observed however, that the inner circle of our model was not completely open (Fig. 12b). To improve control over crosslinking and avoid LAP-concentration dependent cytotoxicity [36], we implemented photoabsorption using food dyes and further refined the formulation by introducing radical scavenging via Vitamin C. By adding Tartrazine (Acid yellow 23, TCI Chemicals, #F0088) at dilutions of 1:500–1:100 (0.01–0.05 % w/v, as reported by Torras et al. [27]) and increasing the accumulated energy dose to 57,2 mJ*s/cm^2^ (20 s) were able to obtain open circular structures with a dilution of 1:150 (0.033 % w/v) delivering the most accurate recapitulation of the original CAD model (Fig. 12c).

*Attention: Optimizing energy dose, photoinitiator, photo absorption and radical scavenging parameters is highly design, feature and material dependent. One set of parameters might work ideally for specific features and orientations, but may not be suitable for other features in the same design. Hence, compromises on quality and specifically adjusting the design and dimensions are a common strategy to find an ideal set of printing parameters. For instance, increasing exposure time to 20 s at 0.1 % or 0.4 % LAP did not improve fidelity further and led to the same overpolymerization or impractical print durations (see* Supplementary Fig. 1*).*

### 8.2 Characterizing print resolution and orientation fidelity in presence of Vitamin C

**Fig. 13:**
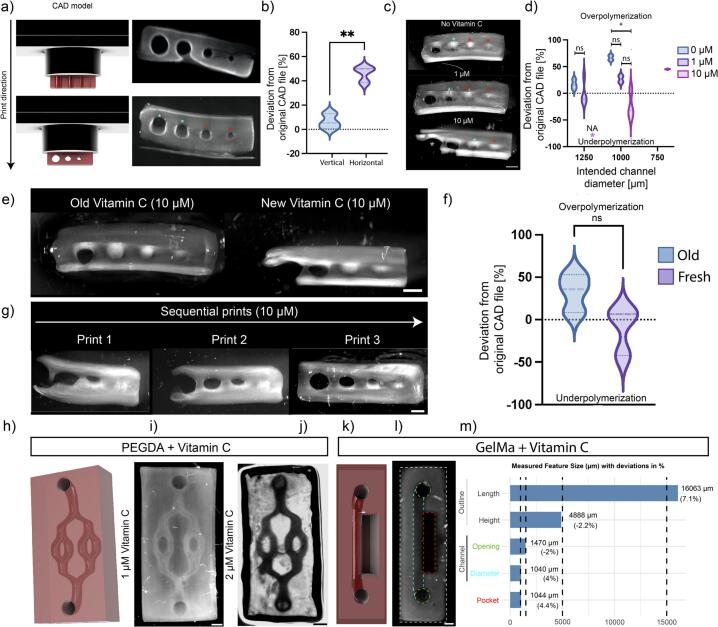
Characterization of resolved printed features with and without Vitamin C. a) Schematic of print orientation of negative feature benchmark and printed benchmarks in vertical (top) and horizontal (bottom) orientation. Cyan aterisks = partly resolved, Red aterisks = not resolved. b) Quantification of the deviations of the channel diameters from the original CAD file in different print orientations. c) Images of horizontally printed benchmarks printed at different concentrations of Vitamin C. Scale bar = 1.5 mm. Cyan aterisks = partly resolved, Red aterisks = not resolved, Magenta aterisks = severely underpolymerized. d) Quantification of the corresponding deviation of channel diameters from the original CAD design at different concentrations of Vitamin C. Magenta aterisk = Data not available due to underpolymerization. e) Negative benchmark printed with old (1 week; left) versus freshly prepared Vitamin C (right). Scale bar = 1.5 mm. f) Quantification of the corresponding deviation of channel diameters from the original CAD design. g) Printed negative feature benchmarks at the same initial concentration of Vitamin C printed consecutively. Scale bar = 1 mm. h) CAD model and bright-field image (i) of a printed chip containing complex channel structures after perfusion with a dyed liquid to demonstrate full perfusability of printed channels printed from PEGDA. Scale bar = 1.5 mm. j) Image of non-perfusable chip printed at a Vitamin C concentration of 1 µM. Scale bar = 2.5 mm. k) Schematic and l) Print of a hydrogel chip (white) containing a single channel (cyan) and two opening (green), next to a pocket (red) printed from GelMA. Scale bar = 2 mm. m) Quantification of measured feature sizes corresponding to the GelMA chip with deviations ranging from −2.2 % to 7,1% (n = 2 chips, repeated measures). (For interpretation of the references to colour in this figure legend, the reader is referred to the web version of this article.)

Since bioprinting is often used to produce microfluidic on-chip models, we assessed the ability of our setup to produce channels of diverse diameters (negative features) at different orientations. We therefore designed a benchmark to test negative features containing channels of 1.5, 1.25, 0.75 and 0.5 mm diameter respectively. While vertical printing of these features obtained fully resolved channels down to the smallest diameters with minimal deviations from the original design (6.38 ± 6.28 %), horizontally printed channels were poorly resolved (Fig. 13a **red asterisks**) with average deviations of 46.10 ± 6.54 % from the original design or not resolved at all (Figs. 13a,b **cyan asterisks**), confirming that resolution depends on print orientation. Adding Vitamin C as a radical scavenger enabled resolving features down to 750 µm diameter in horizontal orientation and influenced channel diameters of bigger channels with a concentration-dependent (0–10 µM) effect (Figs. 13c, d). At the highest concentration (10 µM), we observed severe underpolymerization of the biggest channel. We further explored the use of Vitamin C with this exaggerated concentration (10 µM), to demonstrate effects of radical scavenging in the context of user protocols for bioprinting. Notably, two weeks old Vitamin C compared to freshly made one showed a trend towards overpolymerization, while underpolymerization occurred in samples printed with fresh Vitamin C (Figs. 13e, f). Further, sequential prints from re-used resin revealed subsiding effects of the radical scavenging potential when printing consecutively (Fig. 13g). Together, this demonstrates that the radical scavenging capacity of Vitamin C is reduced over time, necessitating the production of fresh Vitamin C solution as well as fresh bioresin for each print. Moreover, the data confirms that the print orientation has an influence on the printable resolution and that Vitamin C can be added to bioresin formulations to aid printing of small negative features in difficult print orientations. However, channel diameters within the design and concentration of Vitamin C needs to be further optimized to reach optimal results.

### 8.3 Validating print accuracy and freedom of design

Based on the aforementioned findings, the bioresin composition was tested for its capability to produce highly complex architectures such as those found in the human vasculature. We therefore designed a perfusable chip with branching channels of 1 mm diameter (Fig. 1jh**-j**). After further optimization of Vitamin C concentrations for this specific use case (PEGDA 6 % w/v, 0,4 % w/v LAP, increasing Vitamin C from 1 to 2 µM, as well as 20 s to 22 s exposure time/layer; see Fig. 13i**-j**), we obtained perfusable branched vascular network-like structures (Fig. 13j), validating an important use case of bioprinters, to produce vessel-like structures on-a-chip. As a proof-of-concept we printed another chip model comprised of a single channel next to a rectangular pocket from 7.5 % w/v GelMA, 0.4 % w/v LAP, 1 µM Vitamin C and 71.5 mJ*s/cm^2^ (25  s) (Figs. 13j & k). Quantitative analysis of the printed chip features (Fig. 13m) revealed close agreement with CAD specifications, with deviations ranging from –2.2 % to + 7.1 %, confirming the dimensional accuracy of our approach even in soft, natural-derived materials such as GelMA. These results demonstrate the ability of our system to print complex structures from biologically relevant and temperature-sensitive materials in horizontal and vertical orientation and indicate that optimal Vitamin C concentrations might differ between materials and CAD designs, necessitating their optimization in a context-dependent manner. We were able to resolve negative features in horizontal print orientation in the 0.75–1 mm range, which is in line with ranges reported by Kaufmann et al. [15] (1.5 mm) or Breideband et al. [10] (1.5 mm).

By combining radical scavenging through Vitamin C and photoabsorber-based photo-polymerization control, we achieved high structural fidelity, even when using soft, natural-derived bioresins such as GelMA. Our findings also highlight that the scavenging effect of Vitamin C diminishes over time, underlining the importance of fresh resin preparation.

### 8.4 Validating thermal conditions and bioactivity in the presence of Vitamin C

**Fig. 14:**
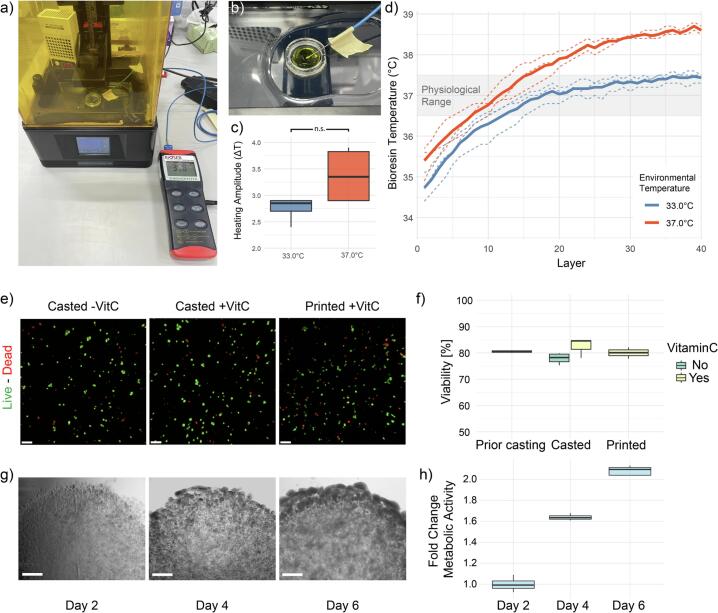
Validating Thermal conditions and bioactivity in the presence of Vitamin C. a) Experimental layout for measuring the photothermal effects, b) with the probe reaching into the bioresin. c) The thermal amplitude normalized to the starting temperature of each individual printing run (n = 4) as boxplots at 33 °C and 37 °C environmental temperature (corresponding to 36/40 °C heater setpoint) respectively. More heat build-up was observed at 37 °C (dT = 3.38 °C) than 33 °C (dT = 2.75 °C) ambient temperature, though results were not statistically significant. Significance was tested with Welch’s *t*-test: p = 0.104; Bayesian posterior 95 % CI = [–0.09, 1.28], see analysis script for quantifications) d) Heat build-up in the bioresin over time (40 layers, 23 mins) depicted as line graphs with bold lines representing the average heating at 33 °C and 37 °C environmental temperature (n = 3). e) Fluorescent images of Live/Dead assays of casted samples with and without Vitamin C and printed samples with Vitamin C (GelMA). Scale bar = 100 µm. f) Quantification of cell viability prior to casting, after casting and after printing. Differences between conditions were statistically not significant according to One-way ANOVA (n = 3). g) Brightfield images of the printed discs including HEK cells over a period of 6 days. Scale bar = 300 µm. h) Alamar Blue readout depicted as fold change metabolic activity and plotted as boxplots (n = 4) over a period of 6 days. (For interpretation of the references to colour in this figure legend, the reader is referred to the web version of this article.)

To better understand how environmental heat and light exposure during printing contribute to the temperature inside the bioresin (photothermal effect[37]), we performed a series of validation experiments using a 10 mm × 2 mm (diameter × height) disc (40 layers á 50 µm, printed in 23 min) and measured the temperature buildup within our bioresin using a BIO TK-8851 device (Fig. 14a). A water solution with the same dye concentration as the bioresin was used to replicate the previous printing conditions (Fig. 14b). We found that the temperature within the resin increased gradually during printing, reaching values beyond the ambient chamber temperature (Fig. 14 **c & d**). This resulted in bioink temperatures outside of the physiological range (∼38.5 °C) when the ambient temperature was set to 37 °C. We observed that lowering ambient temperature by 4 °C resulted in a stabilization of the bioresin temperature within a physiological range of 36.5–37.5 °C without the need for active LCD cooling previously demonstrated by Breideband et al. [10] (Fig. 14d). This heat build-up and strategy to limit it to physiological ranges can become of importance when working with thermally sensitive materials such as GelMA or cells.

Cell viability right after printing with GelMA was determined using single-cell segmentation of imaged Live/Dead assays. Briefly, using Imaris imaging software, the Live and the Dead channel were combined into an “All cells” channel. Single cells were segmented and statistics for cell volume and dead dye intensity were exported. Using a custom R script (available in our repository folder: AnyBio-Analysis), we filtered out debris and defined a dead dye threshold based on separation of populations with different intensity peaks. Subsequently, the percentage of single cells below this threshold was quantified as viability and viability data from cell counting prior to casting was inserted manually. Cell viability post printing was around 80 %, similarly to reported viabilities for other light-based printing approaches [28], and not statistically significantly differing from casted samples (∼85 % viability) (Figs. 14e & f). This indicates that no significant short-term phototoxicity takes place during our casting and printing procedure. Further, the addition of Vitamin C did not have an impact on cell viability immediately after printing, confirming that its use for radical scavenging is cell-friendly short-term. To further assess the effect of Vitamin C and confirm long-term cellular bioactivity in printed constructs, we performed Alamar Blue assays over several days. HEK-cell laden bioresins were printed in discs with GelMA, LAP, and Vitamin C and the fluorescence of Alamar blue solution was measured in phenol red–free conditions to enable accurate readouts. The cells within discs showed continuous division and the formation of larger cell aggregates (Fig. 14g) as well as increasing metabolic activity from day 2 to day 6 (Fig. 14h), indicating that Vitamin C in this formulation and printing context does not impair cellular activity and that our bioprinting setup allows for long-term cell viability and activity post-printing.

Accessible light-based bioprinting platforms require more than just high-resolution printing, they demand compatibility with cell-friendly materials, environmental control and adaptable workflows for optimized bioresins. Our approach uses many off-the-shelf components, lowering technical and financial barriers for its implementation in biologically focused labs. The modular and reusable vat design, based on PVA-molded PDMS, allows printing of both low- and high-viscosity resins without reliance on additional consumables.

Existing mSLA-based bioprinters have already demonstrated valuable modifications that can guide further development [[10], [15]]. For instance, Breideband et al. showed additional environmental control, by integrating CO2 control and active cooling to the LCD screen. Although our printing times were in a similar time range than regular cell culturing and therefore not requiring the addition of CO2, the establishment of gas control for more time consuming prints will likely be a valuable improvement to ensure high cell viability and functionality post-printing. Besides placing the setup in an CO2-regulated environment, e.g. a cell-incubator, one could adjust the bioresin formulation with cell-compatible pH buffers like HEPES (4-(2-hydroxyethyl)-1-piperazineethanesulfonic acid). Further, modular and adjustable build platforms [[10], [15]], could be adapted to a multiwell array with a compatible PDMS-vat shape, enabling parallel construct printing. Together, these modifications could enhance the current AnyBio setup or inspire future conversions.

There remains untapped potential to further improve high-resolution printing in open-source mSLA bioprinting, as light transmission efficiency is fundamentally limited by the LCD screen. Light loss occurs due to internal elements like Thin-Film-Transistors (TFTs), polarizers and electrodes [38], further reduced by irradiance non-uniformity [39], and grayscale-dependent transmittance [27]. DMD-based DLP systems avoid these losses and deliver higher energy per voxel at equal power density [[19], [40]]. Improved LCD panels with higher UV transmission and greater pixel density would reduce exposure times and enhance printing accuracy. For example, Vedhanayagam et al. achieved sub-100 μm resolution by combining a high-resolution mSLA printer with a UV-absorbing resin formulation [20]. Our own strategy follows a similar logic, combining photoabsorbers and radical scavengers to reduce light scattering, prevent overcuring, thus achieving better resolution, while protecting embedded cells. In the future, the introduction of multimaterial printing could also be a valuable addition to enhance the physiological relevance of printed constructs, by for example enabling precise spatial control over the differences in mechanical properties and multi-layering of different materials as found in native tissue [[41], [42]].

### 8.5 Capabilities and limitations of the hardware

#### 8.5.1 Capabilities

• **Easy assembly and accessibility**: No soldering, wiring, or coding required; low total cost (< 350 €) using only off-the-shelf components.
• **High-resolution printing in cell-friendly conditions:** Enables complex feature fabrication (<1 mm) using soft, temperature-sensitive bioresins (e.g. GelMA, PEGDA), including cell-laden hydrogels with high post-print viability (∼ 80 %).
• **Modular vat and platform design**: PDMS-based vat supports both low- and high-viscosity resins; easily adaptable to different printing needs.
• **Reusability and sterility**: Fully reusable vat system eliminates the need for disposable plastic/glass slides or cell culture chambers; allows for easy washing and sterilisation.

#### 8.5.2 Limitations

• **Single-material and single-vat printing**: Current setup is limited to one bioresin at a time, restricting throughput and parallelization.
• **No integrated CO2 or cooling**: Environmental control could be improved via active CO2 supplementation and direct cooling of cell-contacting surfaces.
• **Reduced light transmission in LCD systems**: The LCD screen attenuates curing light, requiring longer exposures compared to DMD systems with equivalent light sources [19,27,[39], [38], [40]].

## Ethics statements

No human or animal experiments were conducted in this work.

## Declaration of Generative AI and AI-assisted technologies in the writing process

During the preparation of this work the authors used ChatGPT-4o in order to improve readability and sentence syntax. After using this tool, the author reviewed and edited the content as needed and takes full responsibility for the content of the published article.

## CRediT authorship contribution statement

**Maj-Britt Buchholz:** Writing – review & editing, Writing – original draft, Visualization, Validation, Methodology, Investigation, Conceptualization. **Nils Bessler:** Writing – review & editing, Writing – original draft, Validation, Methodology, Funding acquisition, Conceptualization. **Anne C. Rios:** Writing – review & editing, Supervision, Resources.

## Declaration of competing interest

The authors declare that they have no known competing financial interests or personal relationships that could have appeared to influence the work reported in this paper.
