# Supplementary figures and images for: AnyBio – An easy off-the-shelf masked stereolithography bioprinter conversion combined with radical-scavenging strategies
Source: HardwareX. 2025 Sep 19;24:e00705. doi: 10.1016/j.ohx.2025.e00705 (PMC12553075; doi:10.1016/j.ohx.2025.e00705)

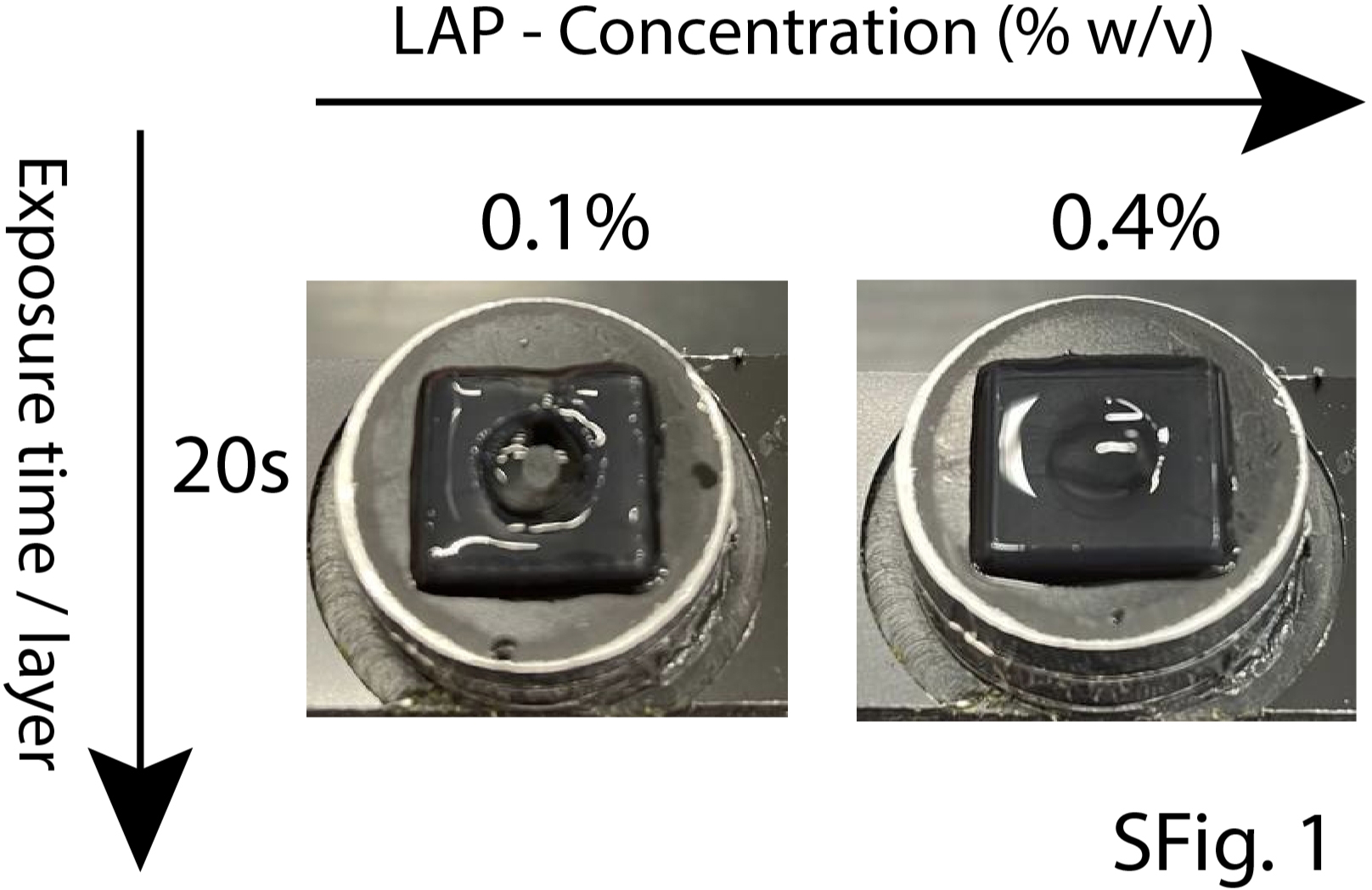

Supplement: Supplementary Fig. 1 [file mmc1.jpg]
